# 5-Chloro-3-ethyl­sulfinyl-2-(4-iodo­phen­yl)-1-benzofuran

**DOI:** 10.1107/S1600536810033581

**Published:** 2010-08-28

**Authors:** Hong Dae Choi, Pil Ja Seo, Byeng Wha Son, Uk Lee

**Affiliations:** a Department of Chemistry, Dongeui University, San 24 Kaya-dong Busanjin-gu, Busan 614-714, Republic of Korea; b Department of Chemistry, Pukyong National University, 599-1 Daeyeon 3-dong, Nam-gu, Busan 608-737, Republic of Korea

## Abstract

In the title compound, C_16_H_12_ClIO_2_S, the 4-iodo­phenyl ring is rotated out of the benzofuran plane by 9.4 (1)°. In the crystal structure, inter­molecular C—H⋯π inter­actions and short inter­molecular I⋯O contacts [3.142 (2) Å] are observed.

## Related literature

For the crystal structures of related 3-ethyl­sulfinyl-2-(4-iodo­phen­yl)-1-benzofuran derivatives, see: Choi *et al.* (2010**a* ▶,b* ▶). For a review on halogen bonding, see: Politzer *et al.* (2007 ▶).
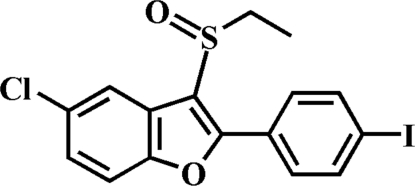


## Experimental

### 

#### Crystal data

C_16_H_12_ClIO_2_S
*M* *_r_* = 430.67
Monoclinic, 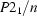

*a* = 11.9782 (3) Å
*b* = 10.4604 (3) Å
*c* = 12.9624 (4) Å
β = 107.827 (1)°
*V* = 1546.16 (8) Å^3^
*Z* = 4
Mo *K*α radiation
μ = 2.38 mm^−1^
*T* = 173 K
0.35 × 0.25 × 0.14 mm

#### Data collection

Bruker SMART APEXII CCD diffractometer
Absorption correction: multi-scan (*SADABS*; Bruker, 2009 ▶) *T*_min_ = 0.514, *T*_max_ = 0.746
14190 measured reflections
3549 independent reflections
3252 reflections with *I* > 2σ(*I*)
*R*_int_ = 0.032

#### Refinement

*R*[*F* ^2^ > 2σ(*F* ^2^)] = 0.025
*wR*(*F* ^2^) = 0.054
*S* = 1.67
3549 reflections
191 parameters
H-atom parameters constrained
Δρ_max_ = 0.75 e Å^−3^
Δρ_min_ = −0.91 e Å^−3^

### 

Data collection: *APEX2* (Bruker, 2009 ▶); cell refinement: *SAINT* (Bruker, 2009 ▶); data reduction: *SAINT*; program(s) used to solve structure: *SHELXS97* (Sheldrick, 2008 ▶); program(s) used to refine structure: *SHELXL97* (Sheldrick, 2008 ▶); molecular graphics: *ORTEP-3* (Farrugia, 1997 ▶) and *DIAMOND* (Brandenburg, 1998 ▶); software used to prepare material for publication: *SHELXL97*.

## Supplementary Material

Crystal structure: contains datablocks global, I. DOI: 10.1107/S1600536810033581/nc2194sup1.cif

Structure factors: contains datablocks I. DOI: 10.1107/S1600536810033581/nc2194Isup2.hkl

Additional supplementary materials:  crystallographic information; 3D view; checkCIF report

## Figures and Tables

**Table 1:** Hydrogen-bond geometry (Å, °) *Cg* is the centroid of the C2–C7 phenyl ring.

*D*—H⋯*A*	*D*—H	H⋯*A*	*D*⋯*A*	*D*—H⋯*A*
C15—H15*A*⋯*Cg*^i^	0.97	3.04	3.750 (3)	131

Symmetry code: (i) 
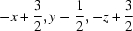
.

**Fig. 1.**
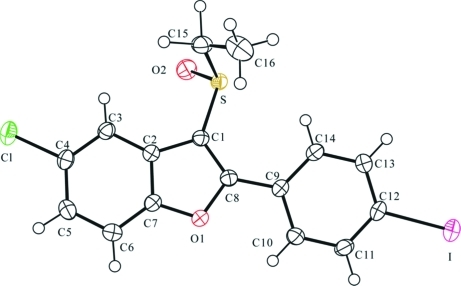
The molecular structure of the title compound with the atom numbering scheme. Displacement ellipsoids are drawn at the 50% probability level. H atoms are presented as a small spheres of arbitrary radius.

**Fig. 2.**
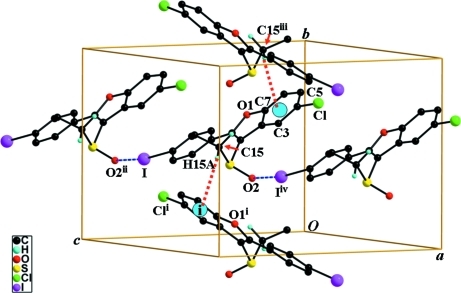
C—H···π and I···O interactions (dotted lines) in the crystal structure of the title compound. Cg denotes the ring centroid. [Symmetry codes: (i) - x + 3/2, y - 1/2, - z + 3/2; (ii) x - 1,y, z; (iii) - x + 3/2, y + 1/2, - z + 3/2; (iv) x + 1, y, z.]

## supplementary crystallographic information

### Comment

As a part of our ongoing studies of the substituent effect on the solid state structures of 3-ethylsulfinyl-2-(4-iodophenyl)-1-benzofuran analogues (Choi *et al.*, 2010*a*,*b*), we report the crystal structure of the title compound (Fig. 1).

The benzofuran unit is essentially planar, with a mean deviation of 0.015 (2) Å from the least-squares plane defined by the nine constituent atoms. The dihedral angle formed by the benzofuran plane and the 4-iodophenyl ring is 9.4 (1)° (Fig. 1). In the crystal structure weak C—H···π interactions between the methylene H atom of the ethyl group and the benzene ring of an adjacent benzofuran ring is observed (Fig. 2 and Tab. 1). In addition, a short I···O contact is observed [I···O2^ii^ = 3.142 (2)Å; C12—I···O2^ii^ = 158.24 (7)°] which indicate a weak interaction (Politzer *et al.*, 2007).

### Experimental

3-chloroperoxybenzoic acid (77%) (202 mg, 0.9 mmol) was added in small portions to a stirred solution of 5-chloro-3-ethylsulfanyl-2-(4-iodophenyl)-1-benzofuran (373 mg, 0.9 mmol) in dichloromethane (30 mL) at 273 K. The mixture was stirred for 5 h at room temperature, washed with saturated sodium bicarbonate solution and the organic layer was separated, dried over magnesium sulfate, filtered and concentrated at reduced pressure. The residue was purified by column chromatography (hexane-ethyl acetate, 1:1 v/v) to afford the title compound as a colorless solid [yield 76%, m.p. 439–440 K; *R*_f_ = 0.56 (hexane-ethyl acetate, 1:1 v/v)]. Single crystals suitable for X-ray diffraction were prepared by slow evaporation of a solution of the title compound in acetone at room temperature.

### Refinement

All H atoms were positioned geometrically and refined using a riding model, with C—H = 0.93 Å for aryl, 0.97 Å for methylene, and 0.96 Å for methyl H atoms. *U*_iso_(H) = 1.2*U*_eq_(C) for aryl and methylene H atoms, and 1.5*U*_eq_(C) for methyl H atoms.

### Crystal data

**Table:**

C_16_H_12_ClIO_2_S	*F*(000) = 840
*M**_r_* = 430.67	*D*_x_ = 1.850 Mg m^−^^3^
Monoclinic, *P*2_1_/*n*	Mo *K*α radiation, λ = 0.71073 Å
Hall symbol: -P 2yn	Cell parameters from 8003 reflections
*a* = 11.9782 (3) Å	θ = 2.6–27.5°
*b* = 10.4604 (3) Å	µ = 2.38 mm^−^^1^
*c* = 12.9624 (4) Å	*T* = 173 K
β = 107.827 (1)°	Block, colourless
*V* = 1546.16 (8) Å^3^	0.35 × 0.25 × 0.14 mm
*Z* = 4	

### Data collection

**Table:**

Bruker SMART APEXII CCD diffractometer	3549 independent reflections
Radiation source: rotating anode	3252 reflections with *I* > 2σ(*I*)
graphite multilayer	*R*_int_ = 0.032
Detector resolution: 10.0 pixels mm^-1^	θ_max_ = 27.5°, θ_min_ = 2.6°
φ and ω scans	*h* = −15→15
Absorption correction: multi-scan (*SADABS*; Bruker, 2009)	*k* = −13→13
*T*_min_ = 0.514, *T*_max_ = 0.746	*l* = −16→15
14190 measured reflections	

### Refinement

**Table:**

Refinement on *F*^2^	Primary atom site location: structure-invariant direct methods
Least-squares matrix: full	Secondary atom site location: difference Fourier map
*R*[*F*^2^ > 2σ(*F*^2^)] = 0.025	Hydrogen site location: difference Fourier map
*wR*(*F*^2^) = 0.054	H-atom parameters constrained
*S* = 1.67	*w* = 1/[σ^2^(*F*_o_^2^) + (0.*P*)^2^] where *P* = (*F*_o_^2^ + 2*F*_c_^2^)/3
3549 reflections	(Δ/σ)_max_ = 0.001
191 parameters	Δρ_max_ = 0.75 e Å^−^^3^
0 restraints	Δρ_min_ = −0.91 e Å^−^^3^

### Special details

**Table:**

Geometry. All esds (except the esd in the dihedral angle between two l.s. planes) are estimated using the full covariance matrix. The cell esds are taken into account individually in the estimation of esds in distances, angles and torsion angles; correlations between esds in cell parameters are only used when they are defined by crystal symmetry. An approximate (isotropic) treatment of cell esds is used for estimating esds involving l.s. planes.
Refinement. Refinement of F^2^ against ALL reflections. The weighted R-factor wR and goodness of fit S are based on F^2^, conventional R-factors R are based on F, with F set to zero for negative F^2^. The threshold expression of F^2^ > 2sigma(F^2^) is used only for calculating R-factors(gt) etc. and is not relevant to the choice of reflections for refinement. R-factors based on F^2^ are statistically about twice as large as those based on F, and R- factors based on ALL data will be even larger.

### Fractional atomic coordinates and isotropic or equivalent isotropic displacement parameters (Å^2^)

**Table:**

	*x*	*y*	*z*	*U*_iso_*/*U*_eq_	
I	0.020729 (12)	0.410793 (14)	0.735141 (13)	0.03083 (6)	
Cl	0.91427 (5)	0.77704 (6)	0.50411 (5)	0.03458 (14)	
S	0.66881 (5)	0.44141 (5)	0.74904 (5)	0.02532 (12)	
O1	0.45082 (12)	0.67491 (14)	0.53906 (12)	0.0240 (3)	
O2	0.75087 (15)	0.37662 (16)	0.69934 (15)	0.0382 (4)	
C1	0.59438 (18)	0.56061 (19)	0.65608 (17)	0.0217 (4)	
C2	0.64728 (18)	0.6403 (2)	0.59295 (17)	0.0215 (4)	
C3	0.76078 (18)	0.6605 (2)	0.58850 (18)	0.0243 (5)	
H3	0.8249	0.6181	0.6349	0.029*	
C4	0.77369 (19)	0.74651 (19)	0.51177 (18)	0.0249 (5)	
C5	0.6793 (2)	0.8095 (2)	0.43905 (19)	0.0287 (5)	
H5	0.6921	0.8647	0.3875	0.034*	
C6	0.5667 (2)	0.7897 (2)	0.44369 (19)	0.0293 (5)	
H6	0.5023	0.8309	0.3965	0.035*	
C7	0.55461 (18)	0.7064 (2)	0.52149 (17)	0.0229 (4)	
C8	0.47620 (18)	0.58480 (19)	0.62118 (17)	0.0220 (4)	
C9	0.37422 (17)	0.5421 (2)	0.65070 (17)	0.0222 (4)	
C10	0.2620 (2)	0.5747 (2)	0.5829 (2)	0.0274 (5)	
H10	0.2541	0.6210	0.5199	0.033*	
C11	0.16345 (19)	0.5382 (2)	0.60939 (19)	0.0290 (5)	
H11	0.0895	0.5625	0.5654	0.035*	
C12	0.17458 (18)	0.4656 (2)	0.70127 (18)	0.0242 (5)	
C13	0.2839 (2)	0.4300 (2)	0.7671 (2)	0.0335 (6)	
H13	0.2911	0.3797	0.8279	0.040*	
C14	0.38293 (19)	0.4693 (2)	0.74239 (19)	0.0320 (5)	
H14	0.4566	0.4467	0.7880	0.038*	
C15	0.7574 (2)	0.5465 (2)	0.8530 (2)	0.0342 (6)	
H15A	0.8221	0.4984	0.9004	0.041*	
H15B	0.7900	0.6136	0.8192	0.041*	
C16	0.6874 (3)	0.6064 (2)	0.9197 (2)	0.0447 (7)	
H16A	0.6264	0.6589	0.8739	0.067*	
H16B	0.7382	0.6579	0.9760	0.067*	
H16C	0.6531	0.5402	0.9516	0.067*	

### Atomic displacement parameters (Å^2^)

**Table:**

	*U*^11^	*U*^22^	*U*^33^	*U*^12^	*U*^13^	*U*^23^
I	0.02630 (9)	0.03230 (10)	0.03857 (11)	−0.00398 (6)	0.01689 (7)	−0.00332 (6)
Cl	0.0289 (3)	0.0336 (3)	0.0473 (4)	−0.0040 (2)	0.0206 (3)	−0.0008 (3)
S	0.0215 (3)	0.0245 (3)	0.0300 (3)	0.0028 (2)	0.0080 (2)	0.0053 (2)
O1	0.0194 (7)	0.0275 (8)	0.0247 (8)	0.0010 (6)	0.0061 (6)	0.0032 (6)
O2	0.0343 (9)	0.0375 (9)	0.0468 (11)	0.0128 (8)	0.0186 (8)	0.0069 (8)
C1	0.0218 (10)	0.0211 (10)	0.0218 (12)	0.0017 (8)	0.0058 (8)	0.0008 (8)
C2	0.0222 (10)	0.0205 (10)	0.0223 (11)	0.0002 (8)	0.0073 (8)	−0.0019 (8)
C3	0.0210 (10)	0.0236 (11)	0.0282 (12)	0.0021 (9)	0.0076 (9)	0.0000 (9)
C4	0.0237 (10)	0.0240 (11)	0.0295 (13)	−0.0030 (9)	0.0120 (9)	−0.0048 (9)
C5	0.0338 (12)	0.0277 (12)	0.0265 (13)	−0.0031 (10)	0.0121 (10)	0.0036 (9)
C6	0.0278 (11)	0.0307 (12)	0.0274 (13)	0.0023 (10)	0.0054 (9)	0.0047 (10)
C7	0.0202 (10)	0.0246 (11)	0.0235 (12)	−0.0010 (8)	0.0062 (9)	−0.0017 (9)
C8	0.0228 (10)	0.0217 (10)	0.0205 (11)	0.0003 (8)	0.0052 (8)	−0.0018 (8)
C9	0.0191 (10)	0.0229 (10)	0.0243 (12)	−0.0004 (8)	0.0061 (8)	−0.0027 (9)
C10	0.0254 (11)	0.0255 (11)	0.0305 (13)	0.0005 (9)	0.0072 (9)	0.0069 (9)
C11	0.0191 (10)	0.0288 (11)	0.0362 (14)	0.0018 (9)	0.0042 (9)	0.0045 (10)
C12	0.0200 (10)	0.0251 (11)	0.0298 (13)	−0.0021 (9)	0.0110 (9)	−0.0055 (9)
C13	0.0288 (12)	0.0459 (15)	0.0280 (14)	0.0019 (11)	0.0121 (10)	0.0084 (11)
C14	0.0198 (11)	0.0482 (15)	0.0274 (13)	0.0027 (10)	0.0063 (9)	0.0059 (11)
C15	0.0295 (12)	0.0372 (13)	0.0291 (14)	−0.0065 (11)	−0.0012 (10)	0.0073 (10)
C16	0.0638 (19)	0.0336 (14)	0.0331 (15)	−0.0055 (13)	0.0093 (13)	−0.0016 (11)

### Geometric parameters (Å, °)

**Table:**

I—C12	2.101 (2)	C6—H6	0.9300
I—O2^i^	3.1422 (17)	C8—C9	1.458 (3)
Cl—C4	1.746 (2)	C9—C14	1.388 (3)
S—O2	1.4928 (17)	C9—C10	1.404 (3)
S—C1	1.773 (2)	C10—C11	1.380 (3)
S—C15	1.810 (2)	C10—H10	0.9300
O1—C7	1.371 (2)	C11—C12	1.384 (3)
O1—C8	1.384 (2)	C11—H11	0.9300
C1—C8	1.371 (3)	C12—C13	1.378 (3)
C1—C2	1.444 (3)	C13—C14	1.382 (3)
C2—C7	1.392 (3)	C13—H13	0.9300
C2—C3	1.394 (3)	C14—H14	0.9300
C3—C4	1.385 (3)	C15—C16	1.513 (4)
C3—H3	0.9300	C15—H15A	0.9700
C4—C5	1.396 (3)	C15—H15B	0.9700
C5—C6	1.384 (3)	C16—H16A	0.9600
C5—H5	0.9300	C16—H16B	0.9600
C6—C7	1.374 (3)	C16—H16C	0.9600
			
C12—I—O2^i^	158.24 (7)	C14—C9—C8	122.93 (19)
O2—S—C1	106.65 (10)	C10—C9—C8	118.68 (19)
O2—S—C15	106.48 (11)	C11—C10—C9	120.4 (2)
C1—S—C15	97.87 (11)	C11—C10—H10	119.8
C7—O1—C8	107.13 (15)	C9—C10—H10	119.8
C8—C1—C2	107.12 (18)	C10—C11—C12	120.1 (2)
C8—C1—S	127.41 (16)	C10—C11—H11	120.0
C2—C1—S	125.09 (16)	C12—C11—H11	120.0
C7—C2—C3	119.10 (19)	C13—C12—C11	120.3 (2)
C7—C2—C1	105.36 (18)	C13—C12—I	121.68 (17)
C3—C2—C1	135.5 (2)	C11—C12—I	118.04 (15)
C4—C3—C2	116.9 (2)	C12—C13—C14	119.8 (2)
C4—C3—H3	121.5	C12—C13—H13	120.1
C2—C3—H3	121.5	C14—C13—H13	120.1
C3—C4—C5	123.1 (2)	C13—C14—C9	121.1 (2)
C3—C4—Cl	118.77 (17)	C13—C14—H14	119.4
C5—C4—Cl	118.11 (17)	C9—C14—H14	119.4
C6—C5—C4	119.9 (2)	C16—C15—S	112.07 (18)
C6—C5—H5	120.0	C16—C15—H15A	109.2
C4—C5—H5	120.0	S—C15—H15A	109.2
C7—C6—C5	116.7 (2)	C16—C15—H15B	109.2
C7—C6—H6	121.6	S—C15—H15B	109.2
C5—C6—H6	121.6	H15A—C15—H15B	107.9
O1—C7—C6	125.38 (19)	C15—C16—H16A	109.5
O1—C7—C2	110.43 (18)	C15—C16—H16B	109.5
C6—C7—C2	124.19 (19)	H16A—C16—H16B	109.5
C1—C8—O1	109.93 (18)	C15—C16—H16C	109.5
C1—C8—C9	136.1 (2)	H16A—C16—H16C	109.5
O1—C8—C9	113.98 (17)	H16B—C16—H16C	109.5
C14—C9—C10	118.39 (19)		
			
O2—S—C1—C8	132.8 (2)	S—C1—C8—O1	−172.77 (15)
C15—S—C1—C8	−117.3 (2)	C2—C1—C8—C9	−177.9 (2)
O2—S—C1—C2	−39.1 (2)	S—C1—C8—C9	9.1 (4)
C15—S—C1—C2	70.8 (2)	C7—O1—C8—C1	0.7 (2)
C8—C1—C2—C7	−1.2 (2)	C7—O1—C8—C9	179.31 (17)
S—C1—C2—C7	172.11 (16)	C1—C8—C9—C14	9.4 (4)
C8—C1—C2—C3	−179.9 (2)	O1—C8—C9—C14	−168.7 (2)
S—C1—C2—C3	−6.6 (4)	C1—C8—C9—C10	−170.0 (2)
C7—C2—C3—C4	0.0 (3)	O1—C8—C9—C10	11.9 (3)
C1—C2—C3—C4	178.6 (2)	C14—C9—C10—C11	2.0 (3)
C2—C3—C4—C5	−1.6 (3)	C8—C9—C10—C11	−178.6 (2)
C2—C3—C4—Cl	179.06 (16)	C9—C10—C11—C12	−2.1 (3)
C3—C4—C5—C6	1.8 (3)	C10—C11—C12—C13	0.4 (3)
Cl—C4—C5—C6	−178.83 (18)	C10—C11—C12—I	−178.92 (17)
C4—C5—C6—C7	−0.4 (3)	O2^i^—I—C12—C13	−172.72 (15)
C8—O1—C7—C6	177.6 (2)	O2^i^—I—C12—C11	6.6 (3)
C8—O1—C7—C2	−1.5 (2)	C11—C12—C13—C14	1.4 (4)
C5—C6—C7—O1	179.7 (2)	I—C12—C13—C14	−179.32 (18)
C5—C6—C7—C2	−1.3 (3)	C12—C13—C14—C9	−1.5 (4)
C3—C2—C7—O1	−179.39 (19)	C10—C9—C14—C13	−0.2 (4)
C1—C2—C7—O1	1.6 (2)	C8—C9—C14—C13	−179.6 (2)
C3—C2—C7—C6	1.5 (3)	O2—S—C15—C16	−172.90 (17)
C1—C2—C7—C6	−177.5 (2)	C1—S—C15—C16	77.08 (19)
C2—C1—C8—O1	0.3 (2)		

Symmetry codes: (i) *x*−1, *y*, *z*.

### Hydrogen-bond geometry (Å, °)

**Table:**

Cg is the centroid of the C2–C7 phenyl ring.

**Table:**

*D*—H···*A*	*D*—H	H···*A*	*D*···*A*	*D*—H···*A*
C15—H15A···Cg^ii^	0.97	3.04	3.750 (3)	131.

Symmetry codes: (ii) −*x*+3/2, *y*−1/2, −*z*+3/2.
